# NERVE: New Enhanced Reverse Vaccinology Environment

**DOI:** 10.1186/1472-6750-6-35

**Published:** 2006-07-18

**Authors:** Sandro Vivona, Filippo Bernante, Francesco Filippini

**Affiliations:** 1 Molecular Biology and Bioinformatics (MOLBINFO), Department of Biology, University of Padua, viale G. Colombo 3, 35131 Padova, Italy

## Abstract

**Background:**

Since a milestone work on *Neisseria meningitidis *B, Reverse Vaccinology has strongly enhanced the identification of vaccine candidates by replacing several experimental tasks using *in silico *prediction steps. These steps have allowed scientists to face the selection of antigens from the predicted proteome of pathogens, for which cell culture is difficult or impossible, saving time and money. However, this good example of bioinformatics-driven immunology can be further developed by improving *in silico *steps and implementing biologist-friendly tools.

**Results:**

We introduce NERVE (New Enhanced Reverse Vaccinology Environment), an user-friendly software environment for the *in silico *identification of the best vaccine candidates from whole proteomes of bacterial pathogens. The software integrates multiple robust and well-known algorithms for protein analysis and comparison. Vaccine candidates are ranked and presented in a html table showing relevant information and links to corresponding primary data. Information concerning all proteins of the analyzed proteome is not deleted along selection steps but rather flows into an SQL database for further mining and analyses.

**Conclusion:**

After learning from recent years' works in this field and analysing a large dataset, NERVE has been implemented and tuned as the first available tool able to rank a restricted pool (~8–9% of the whole proteome) of vaccine candidates and to show high recall (~75–80%) of known protective antigens. These vaccine candidates are required to be "safe" (taking into account autoimmunity risk) and "easy" for further experimental, high-throughput screening (avoiding possibly not soluble antigens). NERVE is expected to help save time and money in vaccine design and is available as an additional file with this manuscript; updated versions will be available at .

## Background

Reverse Vaccinology (RV) is one of the best examples of how Bioinformatics can boost Molecular Immunology. The conventional approach to design vaccines requires pathogen's cultivation and dissection of its main components before testing their ability to elicit protective immunity. RV's novelty consists in starting the search for immunogenic antigens from *in silico *analyses of the pathogen's genome instead of culturing the microorganism [1]. This allows scientists to save time and money while facing pathogens for which cell culture is difficult or impossible. RV potentially permits researchers to select, in addition to most *in vivo *expressed antigens (the easiest ones to purify), any protein encoded by the genome of a pathogen. RV is less helpful with eucaryotes due to complexities of cellular and tissue organization, and it is more effective with prokaryotes, extra- and intracellular. Indeed, the production of specific antibodies can boost immunity not only against extracellular pathogens, usually controlled by Th2-polarized responses, but also against either obligate or facultative intracellular ones, usually controlled by Th1-polarized responses. Even these latter pathogens are susceptible to humoral immunity during the extracellular phases of their infectious cycle and are made vulnerable by antibody cross-linking that modifies the intracellular milieu through signaling [2,3].

The extracellular pathogen, *Neisseria meningitidis *serogroup B, stands as a milestone for RV. From its genome, Pizza and coworkers selected 570 out of 2158 predicted ORFs for protein expression as those meant to be new, surface-exposed antigens [4]. 350 ORFs were successfully cloned, expressed in *Escherichia coli *and tested in a variety of assays. In the end, five proteins were found to satisfy criteria of surface exposure, conservation/expression in all strains as well as a significant titer in serum bactericidal assays. This work induced further research of other pathogens in the same way: *Porphyromonas gingivalis *[5], *Streptococcus pneumoniae *[6], *Chlamydia pneumoniae *[3], *Bacillus anthracis *[7] and group B streptococci [8]. Enhancing such a genome-based approach with a trascriptome-based one – through the use of DNA microarrays – shows whether, when and to what extent antigens are expressed, thus identifying those highly expressed during infection processes [9]. Finally, proteomics, although expensive and laborious, can verify *in silico *prediction of membrane composition [10].

We focused on the selection of the *in silico *vaccine candidates (VCs) from the list of predicted proteins to address two issues: the possibility (i) of automatizing the process with new criteria of analyses and (ii) of reducing the percentage of VCs to less than the 20–30% reported until now [11]. Indeed, the real goal of *in silico *selection is to choose the minimal number of VCs sufficient to find protective antigens (PAs) during experimental phases, thus designing a vaccine that conserves time and money. Therefore, it has to be stressed that obtaining a high recall of PAs in a very small number of selected VCs is more convenient than trying to find all possible PAs. According to this approach it is more productive to select the PAs that will most likely be easily expressed. This reduces the risk of experimental troubles. The main cause of failed cloning and expression of 250 out of 600 VCs from *Neisseria meningitidis *B was the presence of more than one transmembrane spanning region (TM) [12]. Thus, we decided to have no more than two predicted TMs as an *a priori *requirement. Of course, viable VCs can be missed because of such a filter; the extracellular loops of multispanning membrane proteins can actually be significant targets especially if reasonably large. Hence, one may focus on fragments likely to be exposed and accessible to antibodies rather than the entire protein in order to overcome eventual cloning and expression problems. Although such a "local" approach is reasonable, we designed the software to select entire antigens for experimental challenges rather than hazarding fine predictions that may not correspond to *in vivo *conditions.

Choosing such a conservative way to face vaccine design inevitably implies missing some PAs, but this is a small price to reach a valuable compromise. Even selecting only surface antigens may imply missing non-surface PAs. Yet discarding non-surface-exposed predicted antigens, thereby forwarding further bioinformatic analyses on selected ones, proved successful. However, to mimimize the risk of loosing good VCs, we stored up information concerning analytical steps for each sequence without distinction: the final output presents only selected VCs, but information concerning all other proteins is retained. VCs are presented with corresponding integrated analyses. In order to "rationalize" selection step, we took into account the importance of avoiding antigens that can potentially cause autoimmunity in man [13]. Since Major Hystocompatibility Complex (MHC) ligands can be really short (as few as eight residues); this problem may rise also from antigens sharing weak global similarity with host proteins. Addressing such a question on a proteomic scale would make manual management of bioinformatic analyses impossible.

We report here on a new, fully automated RV system, developed to predict best VCs from bacterial proteomes (inferred by completely sequenced genomes and publicly available) and to manage and show data by user-friendly output.

## Implementation

**Figure 1:**
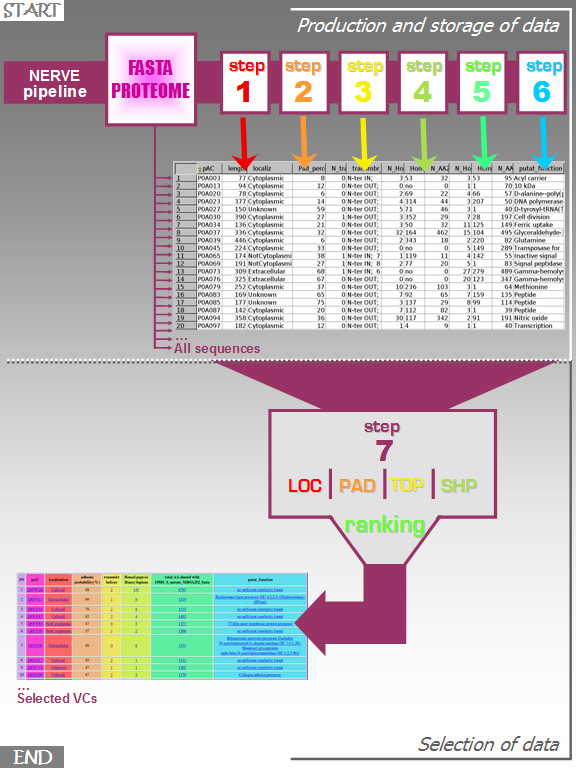
**NERVE software pipeline**. The process can be divided into two parts: data production and storage (top) and data selection (bottom). Six different scripts screen the entire proteome to mine and infer information that flows into a MySQL table. A seventh script uses four filters (LOC, localization; TOP topology; PAD, probability of being adhesin; SHP, similarity to human proteins) and analyzes values created by steps 1 through 5 to select and rank VCs that are then presented in a html table with links to relevant data.

NERVE software pipeline is presented in figure 1. NERVE is composed of eight Perl scripts. A script named "NERVE" leads the user through text-interface configuration. Once this is successfully completed, the script starts and manages the whole process that can be roughly divided in two parts: the first in which all data is produced and stored and the second in which a restricted part of this data is selected. The acquisition of data is performed by six different analytical steps, each forwarded by a special script and each in turn screens the whole proteome indistinctly. All the information produced is stored both in a MySQL table and in text files organized in special subdirectories. Once production and storage of data is completed, another script selects a restricted number of entries from the MySQL table (restriction) and ranks them in a user-friendly html table (ranking). Restriction is performed by four integrated filters and is based on values created by steps 1 to 4. Filters LOC and TOP select non cytoplasmic antigens containing no more than two TMs (NERVE *a priori *requirements). Subsequently, based on empirical threshold values, filters PAD and SHP discard antigens that show a low probability of being adhesins and/or a significant similarity to human proteins. To set these values, NERVE was tuned on ten entire proteomes containing known immunogens; we chose the pair of threshold values that gave the best compromise between restrictivness and immunogen recall. After restriction, NERVE uses values created by step 5 to rank the antigens extracted by the filters. An html table presents this restricted and ranked pool of VCs, showing in six columns the values created by the analytical steps. Entries and data are linked to the corresponding textfiles, thus providing the user with complete information.

## Results and discussion

The system we created predicts the best VCs starting from the flatfile proteome of a prokaryotic pathogen. It forwards six proteome-wide analyses that mine and save data in text files and in an automatically generated MySQL table. The table will contain as many records of extracted information as the number of sequences in thirteen fields. The best VCs are finally selected and are shown in a user-friendly html table reporting seven out of the thirteen fields, linked to the textfile-information that they summarize.

The first proteome scannings assign each sequence three predictions: (i) subcellular localization, (ii) adhesin probability and (iii) topology, using the algorithms PSORTb 2.0 [14], SPAAN [15] and HMMTOP [16] respectively. Indeed, surface-exposed proteins such as outer membrane proteins and especially adhesins, are ideal targets for vaccine development [4,17]. At the same time, the presence of more than one TM in many of these VCs, proved problematic in cloning and expression phases of RV (see introduction) [12]. This clearly shows how predicting number of TMs is potentially a crucial step in saving time and money for subsequent experimental tests. The fourth step addresses the problem of sharing similarity regions between pathogen and host proteins. It is well known that in vaccine development this can either cause low immune response/tolerance or autoimmunity [13]. We have chosen the algorithm BLAST to compare each pathogen's sequence as a query against the human proteome [18]. Local alignment is suitable for this task because potential MHC ligands – the ones we search for to help predict potential interferences (tolerance or autoimmunity) with the immune system – can be very short (~9 residues) [19]. Thus, the script scans alignments, extracting each sequence fragment (shared peptide, SP) that shows no more than three "positives" (compatible substitutions) and one "mismatch" (not compatible substitution) per nine-residues (minimal length required) window. In this way we not only take into account MHC II, commonly involved in presentation of exogenous antigens, but also MHC I, involved in cross-presentation [20,21]. These settings can be changed at the start of the manager programme according to the user's preferences. Once an SP is found, corresponding bacterial and human sequences are reported with their position in the protein and the available description for the human entry in the MySQL table and the text file. Once this fourth step is complete, each pathogen's sequence is assigned to a file, named as its accession, and sent to four new fields in the MySQL database. The first two of these fields show query length and number of mined SPs. The third one reports four features for each SP: position, amino acid sequence, occurrence and "MHC ligand". This last feature is expressed as either "positive" or "negative" depending on the results from a screening of human MHC ligands derived from the database MHCPEP [21]. These MHC ligands do not necessarily correspond to T-epitopes. It would be more advantageous to use a T-epitope prediction tool, but as of now there is no such tool available for standalone use. In addition, since the number of unknown MHC ligands is possibly high, "positive" is a "necessary-but-not-sufficient" condition, whereas "negative" stands as an absence of evidence rather than an evidence of absence. The fourth field reports the aggregate number of amino acid residues from all peptides.

**Figure 2:**
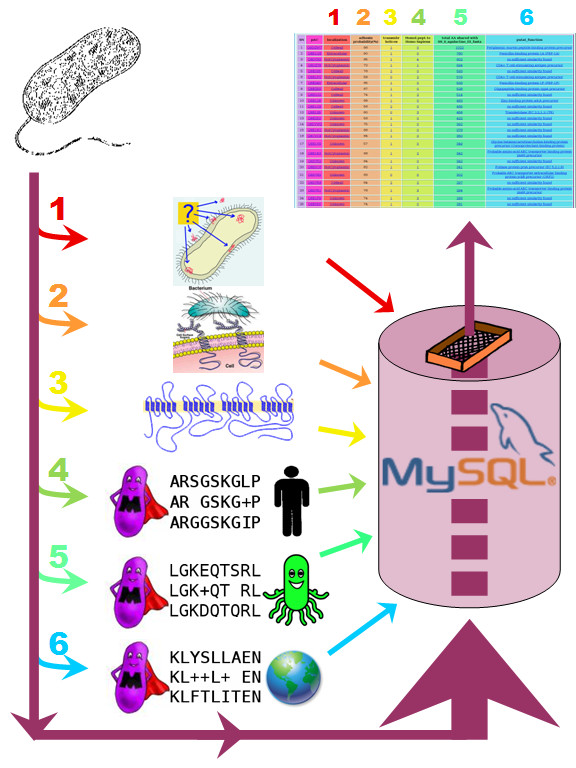
**Flow-chart of NERVE working process**. Amino acid sequences from the whole bacterial proteome undergo six analytical steps: prediction of subcellular localization (1), calculation of probability of being adhesin (2), identification of TM domains (3), comparison to the proteome of *Homo sapiens *(4) and to that of a pathogen selected by the user (5), assignment of a putative function (6). Each of these steps stores data mined in an SQL database. After filtering and ranking, the best VCs are presented in a user-friendly html table (see figure 1 and Results and Discussion for details).

The fifth step allows the user to compare the pathogen's proteome to any other from a different strain/serogroup. Indeed, inferring conservation of each sequence may be helpful in selecting the best VCs as the more an immunogen is conserved, the more protective the vaccine becomes. The way in which we compare two pathogen proteomes is the same process we use to compare pathogen versus human proteomes; all regions potentially capable of binding MHC ligands are searched for in an analogous way. Although this step is not compulsory, it allows NERVE to rank VCs from most to least represented in the compared prokaryotic proteome in the html table presenting the selection set (see below). When possible the sixth step assigns an homology-driven, function prediction to each VC. For instance, this allows one to focus on a VC that shows strong similarity to either known virulence factors or strong immunogens. NERVE uses BLASTp algorithm to compare each sequence to the Uniprot database. According to Ariel *et al*. (2003) [7], the script adopts these parameters: e-value ≤1e-10 and (alignment extension)/(query length) ≥ 0.8. This allows NERVE to attribute to the sequence the subject's most similar function as a query putative function, reported in a further special MySQL column – if no hits are found, the notice "no sufficient similarity found for function inference" is shown. The putative function is then saved in a special text file with the corresponding score and e-value. This last step completes the MySQL database, which in total contains thirteen fields complete with all the information produced along the six analyses. Although each type of information may be retrieved at any time, the final script presents the best VCs in a user-friendly html table. This displays accession numbers and six out of the eleven MySQL fields, linking them to text files containing summarized data (figure 2).

**Figure 3:**
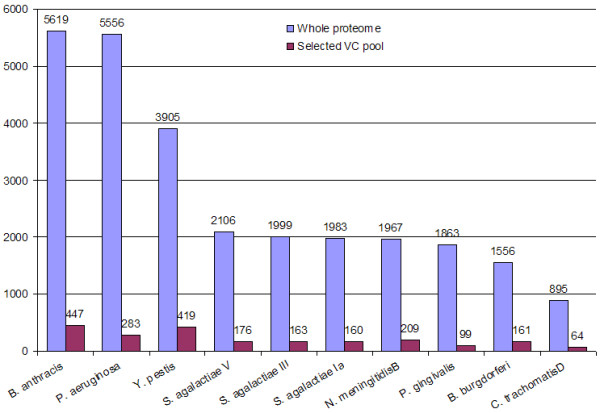
**Data concerning the ten proteomes used for tuning NERVE**. The number of selected VCs is reported beside the overall number of sequences. The average size of the selected VC pools is 8.17% of the proteome (min 5.09%, max 10.73%).

**Figure 4:**
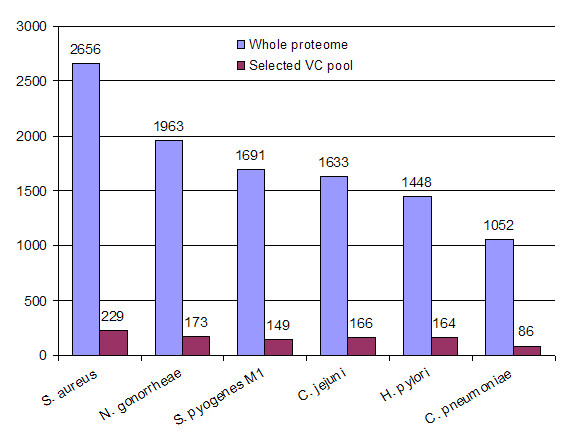
**Data concerning the six proteomes used to test NERVE settings**. The number of selected VCs is reported beside the overall number of sequences. Average size of selected VCs pools is 9.32% of proteome (min 8,17%, max 11,33%).

For final selection of VCs, NERVE uses feature-based, *a priori *requirements to discard proteins that are likely to waste time and money during the experimental tasks (such as those with > 2 TMs) and to exclude non-surface antigens such as cytoplasmic/inner membrane proteins (see introduction for rationale). To further improve final selection efficiency, NERVE considers two characteristics for empirical tuning: the probability that a sequence is an adhesin and the presence of regions common to human proteins. Variation of threshold values for such features along iterative analysis of a known data set (ten proteomes, from both Gram^+ ^and Gram^-^, both extra- and intracellular bacteria) allowed NERVE to define restricted VC pools (average size: 8.17% proteome sequences, figure 3) including a high number (33/42 = 78.6%) of the described PAs that fulfill NERVE *a priori *requirements (see table 1: selected PAs are underlined; PAs not fulfilling NERVE *a priori *requirements are not shown). Settings consistently confirmed themselves as reliably effective – in terms of both PA recall and selection restrictiveness – when tested on six further proteomes (from both Gram^+ ^and Gram^-^, both extra- and intracellular bacteria). In fact, most (22/29 = 75.9%) of the described PAs fulfilling NERVE *a priori *requirements (see table 2: same PAs representation criteria as in table 1) were included in 9.32% whole proteomes average size pools (figure 4).

## Conclusion

RV stands as a turning stone in Vaccinology. It shows how powerful and useful Bioinformatics can be in the post-genomic era. Creating a tool specifically designed to automatize *in silico *steps not only makes RV really available but also time and cost efficient. Indeed, NERVE was conceived to combine automation with an exhaustive treatment of VCs selection task by implementing and integrating six different kinds of analyses. Its modular structure allows further development of new, additional steps as well as the refinement of existing modules. This would improve the compromise between VC selection restrictivness and PA recall. Another goal was to avoid loosing information, thereby giving the user the chance to recover all data mined by NERVE for further investigation. For instance, recovered data regarding shared similarity regions between VCs and human proteins may be of help when taking into account possible occurrences of either tolerance or autoimmunity.

Finally, the NERVE prediction system proved – on a large number of bacterial proteomes – to be reliably effective in selecting very restricted VC pools that are characterized by a high recall (75–80%) of PAs. In particular these results narrow VC pool restriction from the reported 20–30% to an improved 8–9% of proteome sequences. NERVE's attempt to save time and money is also mediated by selection criteria, meant to facilitate crucial experimental steps, the protein cloning and expression phases. Last but not least, NERVE's user-friendly format and easily interpretable output should further aid researchers in designing subunit vaccines against bacterial pathogens.

## Availability and requirements

Project name: NERVE

Home page: 

This software is also available as an additional file [See Additional file 1]

Operating system: Linux (tested distribution: Debian)

Programming language: Perl

Other requirements: Perl 5.8.7 or higher, PSORTb 2.0, SPAAN

License: GNU GPL

Any restrictions to use by non-academics: licence needed

## Abbreviations

MHC = major hystocompatibility complex; ORF = open reading frame; PA = protective antigen; RV = Reverse Vaccinology; SP = shared peptide; TM = transmembrane helix; VC = vaccine candidate.

## Authors' contributions

SV: conception, design, acquisition, analysis and interpretation of data, manuscript drafting; FB: design, manuscript critical reading; FF: conception, analysis and interpretation of data, manuscript drafting. All authors read and approved the final manuscript.

## Figures and Tables

**Table 1:** Tuning NERVE settings on a known dataset including 10 proteomes.

**Pathogen**	**Strain**	**Known protective antigens (non cytoplasmic, ≤2 TMs)**	**Primary accession numbers (Uniprot database)**	**References**
*Bacillus anthracis*	AMES ANCESTOR	PA83, Sap, EA1, AhpC	P13423 P49051 P94217 Q81Y83	[7,22]
*Pseudomonas aeruginosa*	ATCC 15692	OprI, OprF	P11221 P13794	[23–25]
*Yersinia pestis*	CO-92	Caf1, LcrV, YscF	P26948 P21206 Q7ARI0	[26,27]
*Streptococcus agalactiae V*	2603 V/R	Sip, GBS67, GBS80	Q8E2F6 Q8DYR5 Q8E0S9	[8]
*Streptococcus agalactiae III*	NEM316	Sip, GBS67, GBS80, C5a-ase	Q8E7W4 Q8E4C3 Q8E6E4 Q8E4T9	[8]
*Streptococcus agalactiae Ia*	A909	Sip, GBS67, GBS80, C5a-ase, C protein alpha-antigen, C protein beta-antigen	Q3K3Z5 Q3K246 Q3K250 Q3K0M1 Q02192 Q3K3P4	[8]
*Neisseria meningitidis B*	MC58	NspA, PorA, TbpA, TbpB, GNA33, GNA992, NadA, App, GNA1870	Q7DDM2 Q51240 Q9K0U9 Q9K0V0 Q7DDU3 Q7DDJ2 Q9JXK7 Q9JXL6 Q9JXV4	[12]
*Porphyromonas gingivalis*	W83	FimA, HagA, HagB, PG32, PG33	P59914 P59915 Q7MTI5 Q9S3R9 Q9S3R8	[29–32]
*Borrelia burgdorferi*	B31	OspA, OspC, BBI16	P14013 Q07337 O50870	[33–35]
*Chlamydia trachomatis D*	UW-3/Cx	MOMP, OMP3, OMP2	Q46409 P21355 P38006	[36]

**Table 2:** Test of NERVE settings on another dataset including 6 proteomes.

**Pathogen**	**Strain**	**Known protective antigens (non cytoplasmic, ≤2 TMs)**	**Primary accession numbers (Uniprot database)**	**References**
*Staphylococcus aureus*	MW2	Spa, PBP2A, ClfA, collagen-binding, fibronectin-binding, enterotoxin A, alpha-toxin	Q8NXT0 Q8NWP5 P0A0L1 Q8NUH0 Q8NUU7 Q8NX49 Q8NXJ1	[37–39]
*Neisseria gonorrhoeae*	ATCC 700825	PorB, TbpA, TbpB	Q5F5V7 Q5F6Q4 Q5F6Q3	[40,41]
*Streptococcus pyogenes M1*	SF370	Spy0128, Spy0130, Spy0843, Spy1357, Spy1274, Spy1390, Spy0416, Spy2010, Spy2018	P60811 Q7DAL7 Q99ZD7 Q9A0C0 Q9A180 Q9A1S0 Q9A1S2 P58099 Q99XV0	[42]
*Campylobacter jejuni*	NCTC 11168	FlaA, cjaA	P56963 Q9PNV7	[43,44]
*Helicobacter pylori*	J99	CagA, VacA, NAP	Q9ZKW5 Q9ZLT1 Q9ZMJ1	[45]
*Chlamydia pneumoniae*	CWL029	MOMP, Pmp2, ArtJ, HtrA, OmpH-like	P27455 Q9Z3A1 Q9Z869 Q9Z6T0 Q9Z8N7	[46,46]
